# Energetic Extremes in Aquatic Locomotion by Coral Reef Fishes

**DOI:** 10.1371/journal.pone.0054033

**Published:** 2013-01-09

**Authors:** Christopher J. Fulton, Jacob L. Johansen, John F. Steffensen

**Affiliations:** 1 ARC Centre of Excellence for Coral Reef Studies, Research School of Biology, The Australian National University, Canberra, Australian Capital Territory, Australia; 2 ARC Centre of Excellence for Coral Reef Studies, School of Marine and Tropical Biology, James Cook University, Townsville, Queensland, Australia; 3 Marine Biological Section, Department of Biology, University of Copenhagen, Helsingør, Denmark; University of Thessaly, Greece

## Abstract

Underwater locomotion is challenging due to the high friction and resistance imposed on a body moving through water and energy lost in the wake during undulatory propulsion. While aquatic organisms have evolved streamlined shapes to overcome such resistance, underwater locomotion has long been considered a costly exercise. Recent evidence for a range of swimming vertebrates, however, has suggested that flapping paired appendages around a rigid body may be an extremely efficient means of aquatic locomotion. Using intermittent flow-through respirometry, we found exceptional energetic performance in the Bluelined wrasse *Stethojulis bandanensis*, which maintains tuna-like optimum cruising speeds (up to 1 metre s^−1^) while using 40% less energy than expected for their body size. Displaying an exceptional aerobic scope (22-fold above resting), streamlined rigid-body posture, and wing-like fins that generate lift-based thrust, *S. bandanensis* literally flies underwater to efficiently maintain high optimum swimming speeds. Extreme energetic performance may be key to the colonization of highly variable environments, such as the wave-swept habitats where *S. bandanensis* and other wing-finned species tend to occur. Challenging preconceived notions of how best to power aquatic locomotion, biomimicry of such lift-based fin movements could yield dramatic reductions in the power needed to propel underwater vehicles at high speed.

## Introduction

Underwater locomotion is challenging due to the high friction and resistance imposed on a body moving through water and energy lost in the wake during undulatory propulsion [1]–[7]. While aquatic organisms have evolved streamlined shapes to overcome such resistance, aquatic locomotion is still considered a costly exercise [1]–[7]. Indeed, in tunas and other pelagic fishes that cruise the open ocean using tail-powered swimming, we see some of the fastest measured speeds for underwater swimming, but at the cost of some of the highest known rates of aerobic energy consumption during locomotion [5], [6]. However, biomechanical explorations on swimming fish, birds, mammals and reptiles have suggested that flapping paired appendages (fins or flippers) around a rigid-body could be an extremely efficient form of aquatic locomotion at high sustained speeds [7]–[11]. Indeed, recent research on coral reef fishes swimming in this way have shown they can maintain high swimming speeds during everyday activities [8], [12], [13]. Such evidence rivals the paradigm that tail-powered swimming by tuna and other pelagic fishes is the pinnacle of underwater cruising locomotion [5], [6], [13], [14].

Reef fish predominantly swim via the labriform gait, which involves moving solely the pectoral fins for propulsion [12], [13], [14], [15]. Interestingly, these reef fishes display a wide range of pectoral fin shapes, which has been linked to differences in how they move their fins to produce thrust. Coral reef fish with wing-like fins (high aspect-ratio, AR) produce lift-based thrust via figure-eight flapping fin movements, while sister taxa with paddle-shaped fins (low AR) tend to produce resistance-based thrust via a rowing action [7]. Performance studies have indicated such modes translate to different swimming speeds according to the mechanical efficiency and energetic cost of using each form of labriform propulsion [7], [12], [15], [16], [17], with high aspect-ratio species adopting lift-based thrust being the faster swimmers. However, there is currently no empirical comparison of the energetic performance of fishes using these two styles of fin propulsion (hereafter referred to as resistance-based and lift-based labriform swimming). Given that labriform swimming is adopted by a diversity of vertebrates spanning fish to turtles and penguins [9], [10], [15], the underlying efficiency of this mode could help explain the widespread adoption of this form of underwater locomotion.

Integrating new data into a meta-analysis of comparative swimming energetics in bony fishes, we aimed to test the energetic efficiency of resistance- and lift-based labriform swimming for high-speed underwater locomotion. Firstly, we used intermittent flow-through respirometry to measure aerobic energy use during prolonged swimming activity in two species of coral reef fishes from the Great Barrier Reef, Australia: *Stethojulis bandanensis* and *Cheilinus fasciatus*. Chosen for their pectoral fin morphologies (indicated by the pectoral fin AR, which is measured as length of the leading fin edge squared, divided by the fin area taken from digitized fin images following [7], [15], [16]) and similar demersal (reef-associated) microcarnivore lifestyle, these two species span the paddle-shaped (aspect-ratio of 0.86 in *C. fasciatus*) to wing-shaped (2.01 in *S. bandanensis*) fin shape extremes that have previously been linked to the distinct forms of resistance-based and lift-based labriform swimming (e.g. as seen in *Pseudocheilinus octotaenia* with AR of 0.76, and *Gomphosus varius* with AR of 1.75), respectively [7], [12], [15], [16], [17]. Metrics of speed and energetic performance for these two reef fish species were then compared with published values for other non-scombrid and scombrid (tuna-like) fishes to contextualize labriform swimming within the high speed performance of pelagic fishes such as tunas. Performance metrics for this comparison included the rate of increased energy consumption with speed, the gross cost of transporting their body weight one metre per second (GCOT in Joules of energy, [3], [5], [6]), and the optimal swimming speed (*U*_opt_) at which GCOT is minimized for each species [3], [5], [6].

## Materials and Methods

This study was carried out in strict accordance with the protocols approved by The Australian National University Animal Experimentation Ethics Committee (F.BTZ.03.06) for this specific project. All efforts were made to minimize animal suffering through careful collection, handling, and swimming trials based upon the natural rheotaxic behaviour and self-motivation of individuals. Bluelined wrasse *Stethojulis bandanensis* (n = 7, mean ± SE mass  = 15.8±1.1 g, total body length 10.1±0.2 cm, body depth 2.6±0.01 cm, body width 1.4±0.01 cm) and Redbreasted wrasse *Cheilinus fasciatus* (n = 7, 34.2±7.7 g, 12±0.9 cm, 3.8±0.03 cm, 1.6±0.01 cm) were collected from the wild and tested for their swimming performance at the Lizard Island Research Station. Individuals were hand-collected during September 2008 from reefs around Lizard Island, Great Barrier Reef by divers on SCUBA (Self-Contained Underwater Breathing Apparatus) using ultra-fine barrier nets. Transported to the Lizard Island Research Station within 2 hours of capture, fish were maintained in flow-through seawater aquaria at ambient temperatures (27–28°C) and fasted for 36 hours prior to their swimming trial to maximize energy available for swimming and minimize potential respirometer fouling. All individuals were swum within 3 days of capture.

Rate of oxygen consumption (*MO_2_*) by each fish was measured with a computerized, intermittent-flow respirometry system [18], [19]. The clear Perspex respirometer consisted of an 8.3-Litre recirculating flow tank entirely submerged within a 31-Litre aerated seawater bath maintained at ambient temperature (27–28°C). The flow tank could be alternately sealed or flushed with water from the bath via a computer-actuated pump, which allowed high oxygen levels (>80% saturation) to be maintained in the flow tank throughout each swimming trial. The body dimensions and fin spans of all fish (mean ± SE fin span of 4.7±0.03 cm for *S. bandanensis* and 4.6±0.01 cm for *C. fasciatus*) fitted well within the dimensions of the flow tank working section (width 9 cm, depth 11 cm, length 26 cm), which was calibrated by vane-wheel flow probe (Höntzsch GmbH, Waiblingen, Germany) against the voltage output of the propeller motor controller. Honeycomb collimators and curved baffles were used to produce a smooth laminar flow in the working section of the flow tank. Fish were monitored continuously throughout each trial to ensure they swam in this calibrated flow without any use of the walls or corners. Temperature inside the flow tank was maintained at 27.7±0.1°C via a computer-actuated cooling coil housed downstream of the working section. Oxygen partial pressure (*Po_2_*) in the flow tank was measured with an optic-fibre sensor and oxygen meter (Fibox 3, Presens GmbH, Regensburg, Germany) linked to a computer to continuously record *Po_2_* measurements in real time. The respirometer was set to periodically flush the flow tank with aerated water from the bath for 6 minutes, followed by a 2-minute closed mixing period then 7 minutes of closed respirometry when oxygen measurements were taken at a rate of 1 s^−1^ to yield an *MO_2_* at each set flow speed.

Each fish was measured for body depth, width, mass and total length before placement in the respirometer flow tank to calculate solid blocking effects, where they were allowed to acclimate for a minimum of 5 hours at a flow speed of 0.5 total body lengths s^−1^ (4.7–7.9 cm s^−1^). This initial speed provided adequate water mixing whilst allowing the fish to rest on the flow tank bottom without swimming. Oxygen measurements began immediately at the set flushing cycle described above, with these *MO_2_* measurements at 0.5 total body lengths s^−1^ used to calculate standard metabolic rate (SMR, *MO_2_* at zero swim speed). Flow speed was then incrementally increased by 0.5 total body lengths s^−1^ every 15 minutes and *MO_2_* recorded for each set speed. Incremental speed increases were continued until the fish could no longer hold position and became impinged on the downstream grid. To ensure trials measured aerobic locomotion, at the conclusion of each trial, flow speed was reduced to 1 total body length s^−1^ and *MO_2_* was recorded through an additional two flushing cycles. Any post-trial repayments of excessive post-exercise oxygen consumption (EPOC, required to repay any oxygen debt incurred from anaerobic activity in a trial) were measured by comparison of post-trial *MO_2_* against *MO_2_* at 1 total body length s^−1^ at the beginning of the trial. After the fish was removed, the flow tank was resealed and oxygen consumption in the empty respirometer measured to determine background levels, which were subtracted from the *MO_2_* values for the fish swum previously (average ± s.d. backgrounds were 116.7±80.7 mg O_2_ h^−1^, n = 14).

Oxygen consumption rate (*MO_2_*) was determined from the slope of the linear regression of the *Po_2_* decline over time for each measurement cycle, using the formula [8]: *MO_2_* =  *sV*_resp_α where *s* is the slope, *V*_resp_ the volume of the respirometer minus the volume of the fish (calculated from body mass), and α is the solubility of oxygen in seawater. Only measurements with a regression coefficient of determination (*r* ^2^) greater than 0.94 were used in the analyses. SMR was calculated from a frequency histogram of the raw *MO_2_* data within the acclimation period (excluding the first four values immediately after the fish was introduced), by fitting two normal curves to separate the SMR peak, when the fish was at rest, from higher *MO_2_* observations that corresponded with spontaneous activity [20]. Swimming speeds were corrected for solid-blocking effects (minimal for both species, with all fish occupying less than 7% of the flow tank cross-sectional area) following [21] and calculated on-line in real time so that corrected swim speeds were used in the swimming trial settings. The relationship between swimming speed and net cost of swimming was then described using the hydrodynamics-based power equation [3]: Net cost of swimming (*MO_2_* – SMR)  =  a + b *U* ^c^, where a, b and c are constants and *U* is corrected swimming speed in total body lengths per second. This power equation was then transformed following [3] to estimate the optimal swimming speed (*U*_opt_): *U*_opt_  =  [(a+SMR)/(c-1)b]^1/c^, where *U*_opt_ is the speed that minimizes energy expenditure per unit of travel distance. Finally, the gross cost of transport (GCOT, total joules of energy used to move one Newton of body weight one metre) was calculated for each individual by first multiplying total MO_2_ consumption at *U*_opt_ by the conversion factor 14.1 J mg^−1^ O_2_ and then using the equation [3], [5], [6]: GCOT  =  M*O_2_*_(opt)_ (*g U_opt_*)^−1^, where M*O_2_*_(opt)_ is the gross rate of oxygen consumption at *U_opt_* (converted to units of J kg^−1^ hr^−1^), *g* the acceleration due to gravity (9.8 m s^−2^), and *U_opt_* as calculated above (but converted to units of m hr^−1^).

Comparative performance of our study species was examined in the context of a range of bony fishes using published energetic estimates, either taken directly from reported figures in each paper (which often required conversion to the same units stated above) or calculated from fitted equations. Firstly, the exponent in the hydrodynamics-based power equation was used to provide a size-independent measure locomotion efficiency (rate of increase in energy consumption with increasing swimming speed) among a range of fishes. Relative efficiency was calculated by taking the lowest exponent value for a species (1.36 for *S. bandanensis*) and denoting this as 1.00, then diving this by the exponent value for each other species. Secondly, *U*_opt_ for our study species was placed within a log-log plot of *U*_opt_ versus body mass for a range of six scombrid (tuna-like) and seventeen other non-scombrid fishes (including one coral reef fish) following [3] and [6] (Table S1). Finally, the relationship between gross cost of transport (GCOT, J N^−1^ m^−1^) and body mass was used to examine the overall energetic cost incurred by each species swimming at *U*_opt_ following [3].

## Results

**Figure 1:**
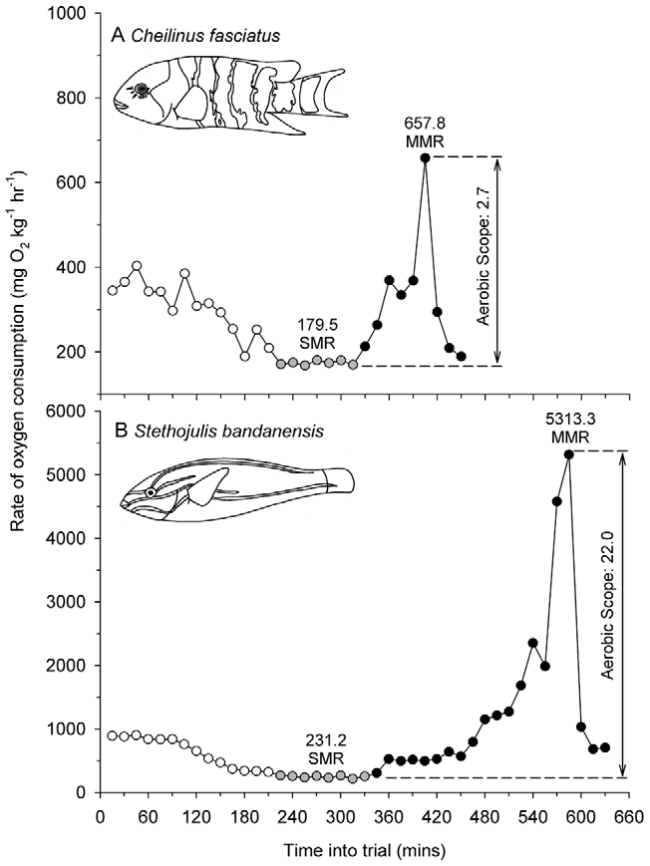
Rate of oxygen consumption against time in a swimming speed trial for (A) Redbreasted wrasse *Cheilinus fasciatus* (11.5 cm total body length) and (B) Bluelined wrasse *Stethojulis bandanensis* (10.5 cm). Factorial aerobic scopes are provided, which is the difference between maximum metabolic rate (MMR) during the trial (highest black dot) and the standard metabolic rate (SMR, grey dots), divided by SMR. Note the different y-axis scales, and rapid *MO_2_* drop on trial completion (last three dots) indicating minimal excessive post-exercise oxygen consumption (EPOC).

**Figure 2:**
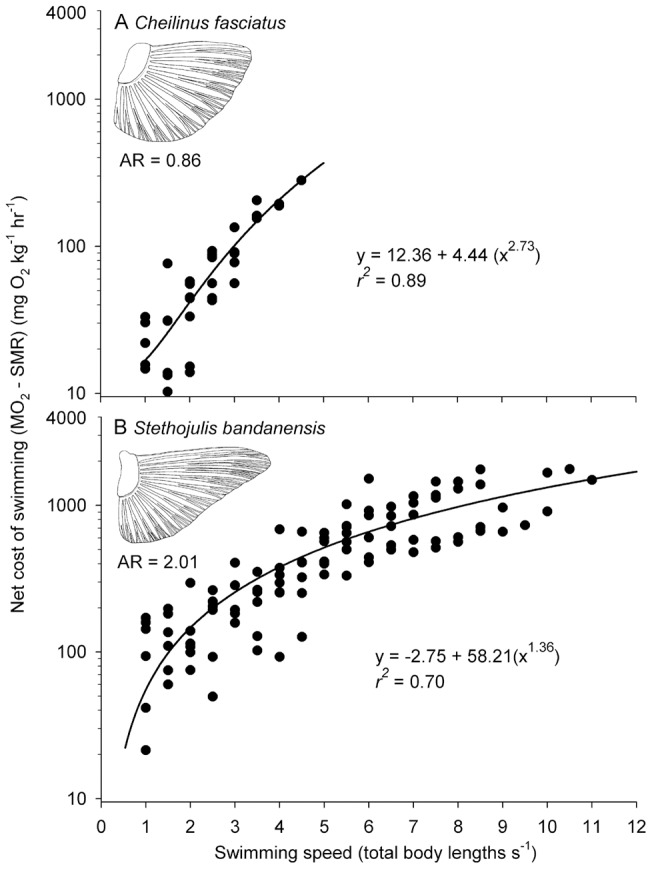
Net cost of swimming for two species of reef fish with alternate pectoral fin shapes, as indicated by the aspect ratio (AR), displayed different increases in oxygen consumption with speed as indicated by the semi-log plots for (A) *Cheilinus fasciatus* and (B) *Stethojulis bandanensis* fitted with the hydrodynamics-based power equation (*MO_2_* – SMR  =  a + b *U* ^c^) [3]. A low exponent value (c) for *S. bandanensis* indicates a very high efficiency of locomotion with increasing speed compared to other bony fishes (Table 1).

**Figure 3:**
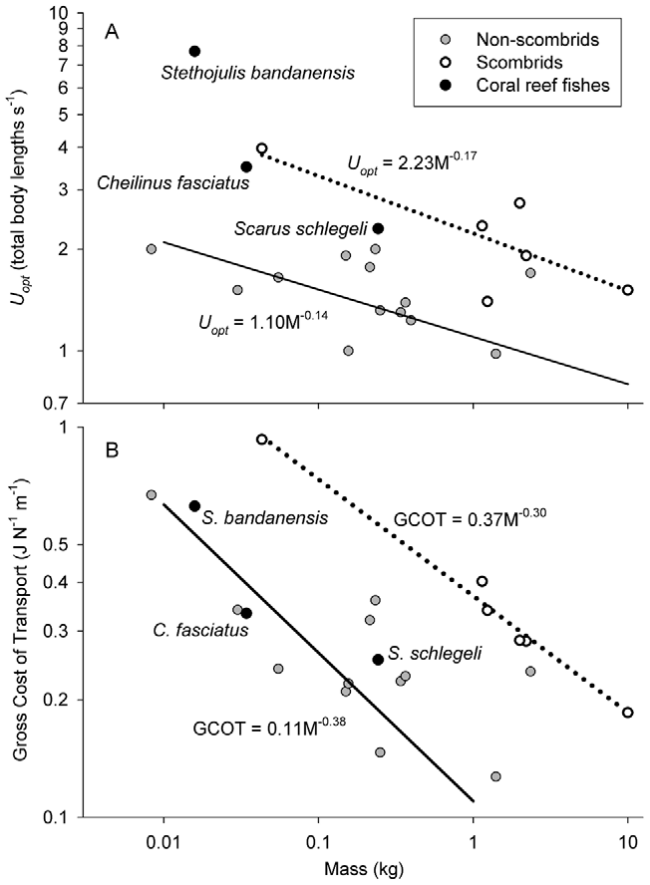
Comparative energetic swimming performance of bony fishes. Three coral reef and sixteen other non-scombrid fishes are presented alongside six scombrid (tuna-like) fishes of varying body mass on log-log plots of (A) Optimum swimming speed (*U_opt_*) and (B) Gross cost of transport (GCOT) incurred by each species at their optimum swimming speed (*U_opt_*). Dotted and solid lines denote mass-*U_opt_* and mass-GCOT power functions [after 3, 6] for scombrid and non-scombrid fishes, respectively (note the range of temperatures encompassed within the underlying data in Table S1 [5], [8], [22]–[24], [26]–[32], which reflect the conditions experienced by each species performing in the wild). Note *S. bandanensis* is well above the scombrid *U_opt_* trend (A, dotted line), but has the same (much lower) GCOT as similar-sized non-scombrid fishes swimming four times slower (B, solid line).

Our study species displayed swimming speeds and energetic profiles at two opposite extremes of the possible performance spectrum for labriform locomotion. At the high extreme, *S. bandanensis* displayed a rate of oxygen consumption ranging from 231 mg O_2_ kg^−1^ hr^−1^ at rest, to over 5,300 mg O_2_ kg^−1^ hr^−1^ at their maximum swimming speed (over 1 metre s^−1^, Fig. 1), which is a factorial aerobic scope 22-fold above resting. By comparison, *C. fasciatus* displayed a factorial aerobic scope of just 2.7-fold (Fig. 1). Moreover, *S. bandanensis* exhibited a very low rate of increase in aerobic metabolism with increasing swimming speed when compared to the related *C. fasciatus* (Fig. 2). As such, *S. bandanensis* displayed the most efficient swimming speed performance profile known for any bony fish swimming via either a labriform or body-caudal gait (represented by the exponent c in Table 1). Indeed, when embedded within the energetic performance of other bony fishes examined to date (23 species from 11 families), *S. bandanensis* is capable of maintaining fast cruising speeds without the high energetic cost incurred by high-speed tunas and other scombrid fishes of similar body size (Table 1 and Fig. 3). When swimming at a speed that minimizes cost of transport, *S. bandanensis* can maintain 7.7 total body lengths s^−1^, which is more than 70% faster than the optimum speeds displayed by other fishes of similar body size (2.0 to 4.5 body lengths s^−1^, Table S1, Fig. 3A). Moreover, *S. bandanensis* can maintain such high optimum swimming speeds without an increase in the gross cost of transport beyond what is seen in a bony fish of similar body mass; tuna-like fishes swimming at similarly high cruising speeds incur up to 40% higher costs of transport than *S. bandanensis* (Fig. 3B). More generally, we see the three labriform-swimming coral reef species examined to date all display faster than expected optimum swimming speeds for their size and cost of transport (Fig. 3).

**Table 1:** Comparative energetic performance of bony fish swimming via pectoral (families Labridae & Scaridae) and caudal (Scombridae, Carangidae, Salmonidae) propulsion, ranked by relative efficiency.

Species	Common name	Family	Factorial Aerobic Scope	Relative Efficiency
*Stethojulis bandanensis*	Bluelined wrasse	Labridae	22.0	1.00
*Thunnus albacares* [6], [22]	Yellowfin tuna	Scombridae	9.0	0.83
*Scarus schlegeli* [8]	Yellowband parrotfish	Scaridae	4.3	0.82
*Trachurus trachurus* [23]	Horse mackerel	Carangidae	9.7	0.53
*Onchorynchus nerka* [6], [24]	Sockeye salmon	Salmonidae	8.2	0.53
*Cheilinus fasciatus*	Redbreasted wrasse	Labridae	2.7	0.50
*Dicentrarchus labrax* [25]	European seabass	Moronidae	2.8	0.50

Temperatures reflected wild conditions and ranged over 20–28°C for these measurements. Published data sources are indicated.

## Discussion

Challenging long-held notions about the costs of underwater locomotion [1]–[4], we find coral reef fishes using labriform locomotion can maintain fast swimming speeds without the elevated cost of transport that has been seen in tunas and other scombrid fishes swimming at such high cruising speeds [5], [6]. Notably, this performance is reflective of their daily activities in the wild, where *S. bandanensis* and other wing-finned species swim at similar or faster speeds (up to 1 metre s^−1^) while they forage across the reef [12], [15], [16]. Understanding the traits that drive such energetic performance in these coral reef fishes can yield important insights for their response to changing environmental conditions, and the technological benefits that could be gained from biomimicry of flapping fin propulsion.

Extreme performance in *S. bandanensis* seems to arise from mechanical efficiency; the comparative performance of *S. bandanensis* and *C. fasciatus* matched predictions based on the relative thrust-producing efficiency of their different pectoral fin shapes and movements [7], [9]–[11], [13], [15]. Such biomechanical and kinematic evidence indicates that *S. bandanensis* achieves high-speed efficiency by sweeping their wing-like (high AR) fins in a figure-eight pattern to produce lift-based thrust on all fin strokes. Rowing paddle-shaped pectoral fins (low AR) in the power-recovery stroke cycle means that *C. fasciatus* produces thrust only half the time [7], [9].

Energetic performance by *S. bandanensis* is brought into extreme relief by comparison with a range of vertebrates spanning multiple families and forms of locomotion. For instance, *S. bandanensis* displayed the widest factorial scope of active metabolism ever seen in a bony fish (up to 22 times above resting, cf. 9 times in Yellowfin tuna [6]) [3], [6], [12], [13], [33], and is comparable to the metabolic scope of active endothermic birds and mammals (typically ranging from 8 to 36 times above resting, [34]). Akin to the high-performing hummingbirds [34], the enormous metabolic scope of this coral reef fish allows maintenance of a wide range of swimming speeds for prolonged time periods. Furthermore, *S. bandanensis* increased their swimming speed with the lowest rate of increased energy consumption seen in a swimming fish. Ultimately, it means this species can optimally travel (i.e. incur a minimum cost of transport) at a speed four times faster than non-scombrid fishes of similar size, without incurring the increased energy use typically seen in tuna-like fishes swimming at this speed. It is interesting to note that other coral reef fishes using labriform swimming also display faster than expected optimal swimming speeds for their size, with similarly low costs of transport, suggesting this may be a generalized feature of pectoral-based propulsion. Collectively, these coral reef fishes adopt oscillatory fin strokes around a rigid-body posture, which is thought to bring hydrodynamic drag down to minimum [1], [8], [14]. When combined with the mechanical efficiency of using a flapping fin stroke to produce lift-based propulsion [1], [7], [9]–[11], [13], [15], the result is very fast sustained speeds in species with wing-like, high aspect-ratio fins. Matching flexibility with efficiency, *S. bandanensis* currently stands out as the highest performing swimmers for their size with respect to measures of scope, optimum swimming speed and energy consumption.

Extremes in energetic performance appear to be linked with ecological extremes in these coral reef fishes. For species with low speed efficiency, such as *C. fasciatus*, we tend to find these fish occupying calm water habitats sheltered from incident wave energy and storms. In contrast, *S. bandanensis* and other wing-finned fish species tend to be in great abundance in habitats subject to highly variable and extreme water flows generated by direct exposure to wave energy [13], [15], [16]. Under such hydrodynamic conditions, high efficiency across a wide range of speeds may be a physiological imperative for species to occupy these challenging, but food-rich habitats [13], [15], [16]. Such extremes may also place these species in good stead for future changes in the marine environment arising from climate change. Both thermal and hydrodynamic conditions appear to be increasingly intense and variable in marine habitats around the globe [35], [36]. With a flexible and wide scope of aerobic metabolism, *S. bandanensis* appears to be equipped with the physiological traits needed to maintain their metabolic delivery of energy across a wide range of activities and hydrodynamic conditions. While *S. bandanensis* may be able to ride through such challenges, species such as *C. fasciatus* and other coral reef fishes of low aerobic scope [8], [33], [37] may require rapid adaptation in order to survive any increases in the intensity and variability of environmental conditions [33]–[37].

Such functional innovation in swimming prompts a rethink of the possibilities and limits to the cost of high speed performance in underwater locomotion. In fishes, we see that relative swimming performance is not necessarily related to their perceived lifestyle, with reef fishes capable of exceptional swimming performance despite their supposed sedentary, reef-associated existence [12]–[16]. Achieved through the use of drag-minimizing rigid-body posture, and exploiting lift-based forces from oscillating fins, these species provide some key lessons for the field of biomimetics. We are just starting to see man-made submersibles with propulsive fins reminiscent of the pectoral fins in coral reef fishes, turtles, marine birds and mammals (e.g. *Madeleine* robot turtle [9]–[11], [38]). By incorporating a lift-based oscillatory fin movement into such technology, dramatic reductions could be achieved in the power needed to propel autonomous underwater vehicles of similar size to the fish and other aquatic animals that use this mechanism [9], [11]–[13], [38].

## Supporting Information

Table S1 **Meta-data for comparative analysis of the energetic swimming performance of reef fishes, scombrid, and non-scombrid fishes.**
(DOC)

## References

[1] Schmidt-Nielson K (1972) Locomotion: energy cost of swimming, flying, and running. Science 177: 222–228. 4557340 10.1126/science.177.4045.222

[2] Vogel S (1994) Life in moving fluids: the physical biology of flow. Princeton: Princeton University Press.

[3] Videler JJ (1993) Fish swimming. New York: Chapman and Hall.

[4] Alexander RM (2005) Models and the scaling of energy costs for locomotion. J Exp Biol 208: 1645–1652. 15855396 10.1242/jeb.01484

[5] Videler JJ, Nolet BA (1990) Costs of swimming measured at optimum speed: scale effects, differences between swimming styles, taxonomic groups and submerged and surface swimming. Comp Biochem Physiol 97A: 91–99. 10.1016/0300-9629(90)90155-l 1982941

[6] Korsmeyer KE, Dewar H (2001) Tuna metabolism and energetics. In: Block B, Stevens ED, editors. Tuna: physiology, ecology and evolution. London: Academic Press. 35–78.

[7] Walker JA, Westneat MW (2002) Kinematics, dynamics, and energetics of rowing and flapping propulsion in fishes. Integr Comp Biol 42: 1032–1043. 21680385 10.1093/icb/42.5.1032

[8] Korsmeyer KE, Steffensen JF, Herskin J (2002) Energetics of median and paired fin swimming, body and caudal fin swimming, and gait transition in parrotfish (*Scarus schlegeli*) and triggerfish (*Rhinecanthus aculeatus*). J Exp Biol 205: 1253–1263. 11948202 10.1242/jeb.205.9.1253

[9] Fish FE (1996) Transitions from drag-based to lift-based propulsion in mammalian swimming. Amer Zool 36: 628–641.

[10] Baudinette RV, Gill P (1985) The energetics of ‘flying’ and ‘paddling’ in water: locomotion in penguins and ducks. J Comp Physiol B 155: 373–380.

[11] Wyneken J (1997) Sea turtle locomotion: mechanics, behaviour, and energetics. In: Lutz PL, Musick JA, editors. The biology of sea turtles. Boca Raton: CRC Press. 165–198.

[12] Fulton CJ (2007) Swimming speed performance in coral reef fishes: field validations reveal distinct functional groups. Coral Reefs 26: 217–228.

[13] Fulton CJ (2010) The role of swimming in reef fish ecology. In: Domenici P, Kapoor BG, editors. Fish locomotion: an eco-ethological perspective. Enfield: Science Publishers. 333–373.

[14] Webb PW (1994) The biology of fish swimming. In: Maddock L, Bone Q, Rayner JM, editors. Mechanics and physiology of animal swimming. Cambridge: Cambridge University Press. 45–62.

[15] Wainwright PC, Bellwood DR, Westneat MW (2002) Ecomorphology of locomotion in labrid fishes. Environ Biol Fish 65: 47–62.

[16] Fulton CJ, Bellwood DR, Wainwright PC (2005) Wave energy and swimming performance shape coral reef fish assemblages. Proc Roy Soc Lond B 272: 827–832. 10.1098/rspb.2004.3029 PMC1599856 15888415

[17] Walker JA, Westneat MW (2002) Performance limits of labriform propulsion and correlates with fin shape and motion. J Exp Biol 205: 177–187. 11821484 10.1242/jeb.205.2.177

[18] Steffensen JF, Johansen K, Bushnell PG (1984) An automated swimming respirometer. Comp Biochem Physiol 79A: 437–440.

[19] Steffensen JF (1989) Some errors in respirometry of aquatic breathers: how to avoid and correct for them. Fish Physiol Biochem 6: 49–59. 24226899 10.1007/BF02995809

[20] Steffensen JF, Bushnell PG, Schurmann H (1994) Oxygen consumption in four species of teleosts from Greenland: no evidence of metabolic cold adaptation. Polar Biol 14: 49–54.

[21] Bell WH, Terhune LDB (1970) Water tunnel design for fisheries research. J Fish Res Bd Can Tech Rep No. 195.

[22] Dewar H, Graham JB (1994) Studies of tropical tuna swimming performance in a large water tunnel. I. Energetics. J Exp Biol 192: 13–31. 9317243 10.1242/jeb.192.1.13

[23] Wardle CS, Soofiani NM, O'Neill FG, Glass CW, Johnstone ADF (1996) Measurements of aerobic metabolism of a school of horse mackerel at different swimming speeds. J Fish Biol 49: 854–862.

[24] Brett JR (1965) The relation of size to rate of oxygen consumption and sustained swimming speed of sockeye salmon (*Oncorhynchus nerka*). J Fish Res Bd Can 22: 1491–1501.

[25] Claireaux G, Couturier C, Groison AL (2006) Effect of temperature on maximum swimming speed and cost of transport in juvenile European sea bass (*Dicentrarchus labrax*). J Exp Biol 209: 3420–3428. 16916977 10.1242/jeb.02346

[26] Clark TD, Seymour RS (2006) Cardiorespiratory physiology and swimming energetics of a high-energy-demand teleost, the yellowtail kingfish (*Seriola lalandi*). J Exp Biol 209: 3940–3951. 16985209 10.1242/jeb.02440

[27] Fitzgibbon QP, Strawbridge A, Seymour RS (2007) Metabolic scope, swimming performance and the effects of hypoxia in the mulloway, *Argyrosomus japonicus* (Pisces: Sciaenidae). Aquacult 270: 358–368.

[28] Herskin J, Steffensen JF (1998) Energy savings in sea bass swimming in a school: measurements of tail beat frequency and oxygen consumption at different swimming speeds. J Fish Biol 53: 366–376.

[29] Sepulveda CA, Dickson KA, Graham JB (2003) Swimming performance studies on the eastern Pacific bonito *Sarda chiliensis*, a close relative of the tunas (family Scombridae). I. Energetics. J Exp Biol 206: 2739–2748. 12847119 10.1242/jeb.00497

[30] Gooding RM, Neill WH, Dizon AE (1981) Respiration rates and low-oxygen tolerance limits in skipjack tuna, *Katsuwonus pelamis* . Fish Bull 79: 31–48.

[31] Graham JB, Lowell WR, Lai NC, Laurs RM (1989) O_2_ tension, swimming velocity, and thermal effects on the metabolic rate of the Pacific albacore *Thunnus alalunga* . Exp Biol 48: 89–94. 2920815

[32] Sepulveda CA, Dickson KD (2000) Maximum sustainable speeds and cost of swimming in juvenile Kawakawa tuna (*Euthynnus affinis*) and chub mackerel (*Scomber japonicus*). J Exp Biol 203: 3089–3101. 11003820 10.1242/jeb.203.20.3089

[33] Johansen JL, Jones GP (2011) Increasing ocean temperature reduces the metabolic performance and swimming ability of coral reef damselfishes. Glob Change Biol 17: 2971–2979.

[34] Bishop CM (1999) The maximum oxygen consumption and aerobic scope of birds and mammals: getting to the heart of the matter. Proc Roy Soc B 266: 2275–2281. 10.1098/rspb.1999.0919 PMC1690458 10629977

[35] Hein AM, Keirsted KJ (2012) The rising cost of warming waters: effects of temperature on the cost of swimming in fishes. Biol Lett 8: 266–269. 22031723 10.1098/rsbl.2011.0885 PMC3297402

[36] Young IR, Zieger S, Babanin AV (2011) Global trends in wind speed and wave height. Science 332: 451–455. 21436400 10.1126/science.1197219

[37] Donelson JM, Munday PL, McCormick MI, Nilsson GE (2011) Acclimation to predicted ocean warming through developmental plasticity. Glob Change Biol 17: 1712–1719.

[38] Long JH Jr, Schumacher J, Livingston N, Kemp M (2006) Four flippers or two? Tetrapodal swimming with an aquatic robot. Bioinsp Biomim 1: 20–29. 17671301 10.1088/1748-3182/1/1/003

