# Supplementary material for: Energetic Extremes in Aquatic Locomotion by Coral Reef Fishes
Source: PLoS One. 2013 Jan 9;8(1):e54033. doi: 10.1371/journal.pone.0054033 (PMC3541231; doi:10.1371/journal.pone.0054033)
Supplement: Table S1 — Meta-data for comparative analysis of the energetic swimming performance of reef fishes, scombrid, and non-scombrid fishes. (DOC) [file pone.0054033.s001.doc]

**Table S1. Meta-data for comparative analysis of the energetic swimming performance** of reef fishes (RF), scombrid (Sc), and non-scombrid (NSc) fishes for which the required swimming energetic parameters have been published from respirometer studies. Note the wide range of temperatures encompassed in this dataset, which reflect the conditions experienced by each species performing in the wild. Italicized *MO_2_* _(opt)_ values have been back-calculated from reported GCOT values [5]. Published data sources are indicated in parentheses.

| Species | Total length (cm) | Body mass (kg) | Temp (°C) | U_opt_  (total body lengths s^-1^) | *MO_2_* _(opt)_ (mg O_2_ kg^-1^ hr^-1^) | GCOT_opt_ (J N^-1^ m^-1^) |
| --- | --- | --- | --- | --- | --- | --- |
| *Stethojulis bandanensis* (RF) | 10 | 0.02 | 28 | 7.90 | 1316 | 0.658 |
| *Cheilinus fasciatus* (RF) | 12 | 0.03 | 28 | 3.38 | 337 | 0.332 |
| *Scarus schlegeli* (RF) [8] | 23 | 0.24 | 27 | 2.30 | 336 | 0.253 |
| *Trachurus trachurus* (NSc) [23] | 31 | 0.36 | 12 | 1.21 | 65 | 0.069 |
| *Onchorynchus nerka* (NSc) [24] | 54 | 1.40 | 15 | 0.98 | 168 | 0.127 |
| *Seriola lalandi* (NSc) [26] | 55 | 2.08 | 20 | 1.20 | 258 | 0.155 |
| *Argyrosomus japonicus* (NSc) [27] | 32 | 0.34 | 22 | 1.30 | 214 | 0.209 |
| *Micropterus salmoides* (NSc) [5] | 23 | 0.15 | 15 | 1.91 | *231* | 0.210 |
| *Pomatomus saltatrix* (NSc) [5] | 25 | 0.23 | 15 | 2.01 | *453* | 0.360 |
| *Morone saxatilis* (NSc) [5] | 25 | 0.21 | 15 | 1.69 | *339* | 0.320 |
| *Melanogrammus aeglefinus* (NSc) [5] | 25 | 0.16 | 10 | 1.00 | *138* | 0.220 |
| *Coregonus clupeaformis* (NSc) [5] | 34 | 0.36 | 17 | 1.35 | *264* | 0.230 |
| *Liza macrolepis* (NSc) [5] | 11 | 0.01 | 29 | 2.00 | *369* | 0.670 |
| *Lepomis gibbosus* (NSc) [5] | 12 | 0.03 | 20 | 1.51 | *154* | 0.340 |
| *Salmo gairdneri* (NSc) [5] | 29 | 0.26 | 15 | 0.96 | *202* | 0.290 |
| *Tilapia nilotica* (NSc) [5] | 21 | 0.08 | 25 | 1.95 | *215* | 0.210 |
| *Onchorynchus nerka* (NSc) [5] | 19 | 0.06 | 15 | 1.65 | *188* | 0.240 |
| *Dicentrarchus labrax* (NSc) [28] | 28 | 0.25 | 20 | 1.32 | 132 | 0.143 |
| *Sarda chiliensis* (NSc) [29] | 47 | 1.19 | 24 | 1.40 | 444 | 0.270 |
| *Thunnus albacares* (Sc) [22] | 42 | 1.10 | 25 | 2.34 | 1030 | 0.418 |
| *Thunnus albacares* (Sc) [22] | 51 | 2.17 | 25 | 1.96 | 676 | 0.270 |
| *Katsuwonus pelamis* (Sc) [30] | 47 | 1.96 | 24 | 2.10 | 736 | 0.298 |
| *Thunnus alalunga* (Sc) [31] | 78 | 10.00 | 15 | 1.51 | 546 | 0.185 |
| *Scomber japonicus* (Sc) [32] | 14 | 0.03 | 24 | 3.93 | 1255 | 0.911 |
| *Euthynnnus affinis* (Sc) [32] | 15 | 0.04 | 24 | 4.46 | 1717 | 1.023 |
